# Chemical chaperone suppresses the antibody aggregation in CHO cell culture

**DOI:** 10.1186/1753-6561-7-S6-P68

**Published:** 2013-12-04

**Authors:** Masayoshi Onitsuka, Miki Tatsuzawa, Masahiro Noda, Takeshi Omasa

**Affiliations:** 1 Institute of Technology and Science, The University of Tokushima, Tokushima, 770-8506, Japan; 2 Graduate School of Advanced Technology and Science, The University of Tokushima, Tokushima, 770-8506, Japan

## Background

Aggregation of therapeutic antibodies could be generated at different steps of the manufacturing process, posing the problem for quality control of produced antibodies. It has been well known that secreted antibodies from recombinant mammalian cells into culture medium can aggregate due to the physicochemical stresses such as media pH and osmolality, cultivation temperature [1,2]. The antibody aggregation during the cell culture process is difficult to suppress because the cell culture conditions for antibody production are generally optimized for cell culture and growth and not for suppressing the aggregate formation. Here we show the novel strategy to suppress the antibody aggregation; application of chemical chaperone to the cell culture process. It is well established that an addition of some cosolutes serves as chemical chaperone to suppress the protein aggregation. Trehalose, non-reducing sugar formed from two glucose units with α-1,1 linkage, is known as an effective chemical chaperone. In this study, we investigated the anti-aggregation effect of trehalose in the culture process of recombinant Chinese hamster ovary cell (CHO) line producing Ex3-humanized IgG-like bispecific single-chained diabody with Fc (Ex3-scDb-Fc). Ex3-scDb-Fc shows the remarkable anti-tumor activity based on anti-EGFR and anti-CD3 bispecificity [3]. However, our in-house results showed that Ex3-scDb-Fc shows aggregation tendency, demonstrating the necessity of developing a bioprocess for suppressing the aggregation of the bispecific diabody.

## Materials and methods

CHO Top-H cell line producing the Ex3-scDb-Fc [4] was cultivated in 500mL Erlenmeyer flask and 2L-glass bioreactor with serum-free medium containing 150mM trehalose. Viable cell densities and antibody concentrations were determined with Vi-Cell XR™ cell viability analyzer (Beckman Coulter) and by ELISA, respectively. Ex3-scDb-Fc was purified with Hi-Trap protein A column (GE Healthcare). 1M Arg-HCl (pH4.2) was used as eluting solution, which make it possible to prevent the aggregation of the antibody in the affinity purification process. Antibody aggregation was analyzed by sephacryl S-300 column (GE healthcare). Solution structure of Ex3-scDb-Fc was assessed by circular dichroism spectroscopy.

## Results and discussion

### Cell culture performance in trehalose containing medium

We cultivated CHO Top-H cell line in 150mM trehalose containing medium. The media osmolalities with and without trehalose (150 mM) were 480 mOsm/kg and 319 mOsm/kg, respectively. Estimated kinetic parameters of cell culture are listed in Table 1. Cell culture in Erlenmeyer flasks demonstrated that cell growth was strongly affected by trehalose; the specific cell growth rate and the maximum cell density were decreased compared to those in the absence of trehalose. On the other hand, both the specific antibody production rate and volumetric production were largely enhanced by trehalose addition. The results in Erlenmeyer flask mentioned above were reproduced in 2L-glass bioreactor culture. Observed properties of the cell culture in the presence of trehaose, suppressed cell growth and enhanced antibody production, were similar to those reported for mammalian cell cultures under hyperosmotic condition [5], although the underlying mechanisms responsible for the enhanced antibody production are largely unknown.

**Table 1:** Kinetic parameters of cell culture in Erlenmeyer flasks.

	Specific growth rate (μ; ×10^-2 ^1/h)	Specific antibody production rate (ρ_Ab_; pg/cell/day)
Without trehalose	3.07 ± 0.18 ^a^	0.39 ± 0.02 ^a^
150mM trehalose	1.51 ± 0.04 ^a^	1.55 ± 0.03 ^a^

^a ^Mean ± S.D. (n = 3).

### Anti-aggregation effects by trehalose during the cell culture process

**Figure 1:**
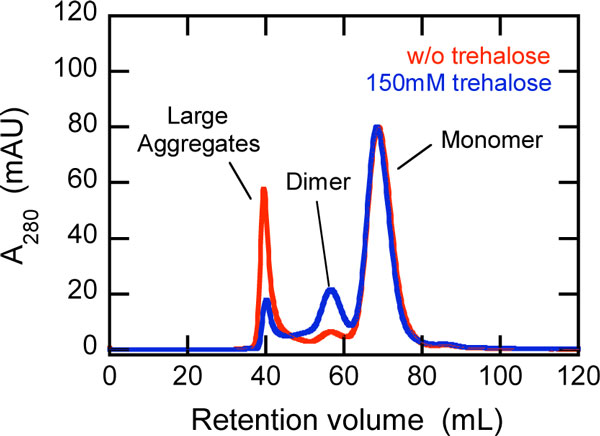
**Size-exclusion chromatography showing the aggregation status of Ex3-scDb-Fc**.

The scDb-Fc was purified from the culture supernatant by protein A affinity chromatography, and the aggregation states were analyzed by size exclusion chromatography. We observed the 3 states of scDb-Fc, monomer, dimer, and large aggregates, which were included in the culture supernatant when harvested (Figure 1). The peak area of the large aggregates in the presence of trehalose was one-third that in the absence of trehalose, indicating that trehalose suppressed the formation of large aggregates in the CHO cell culture. Circular dichroism (CD) spectroscopy showed that the large aggregates were misfolded state with non-native β-strand. Trehalose is expected to suppress the accumulation of misfolded state and the intermolecular interactions leading to the aggregate formation in cell culture.

## Conclusions

We demonstrated the potential application of chemical chaperon in the culture of antibody-producing mammalian cells. Trehalose can be incorporated in the culture media for CHO cells, and can suppress the antibody aggregation, especially high-order aggregates. In addition, trehalose may be involved in the enhancement of antibody production.
